# Quantum-Kernel Learning for Subject-Independent EEG Emotion Recognition with Spectral-Connectivity Features

**DOI:** 10.3390/s26216840

**Published:** 2026-10-29

**Authors:** Gaochao Cui, Yutaka Tokuda, Hideaki Touyama

**Affiliations:** 1 Department of Information Systems Engineering, Faculty of Information Engineering, Toyama Prefectural University, Imizu 939-0398, Japan; touyama@pu-toyama.ac.jp; 2 Independent Researcher, Higashimatsuyama 355-0028, Japan

**Keywords:** electroencephalography, EEG, emotion recognition, quantum machine learning, quantum kernel, differential entropy, phase-locking value, DEAP, support vector machine

## Abstract

Electroencephalography (EEG)-based emotion recognition remains challenging under subject-independent evaluation because of substantial inter-subject variability. This study investigates the feasibility of state-fidelity-based quantum-kernel learning for subject-independent valence and arousal recognition using differential entropy (DE) and phase-locking value (PLV) features. Using the DEAP dataset, DE and PLV features were extracted and evaluated individually and in combination, with subject-wise normalization and F-score feature selection applied within the evaluation framework. Six features were retained for valence and eight for arousal. The selected features were encoded using a Qiskit ZFeatureMap, and the fidelity-based quantum kernel was evaluated through statevector simulation on a classical computer. Quantum-kernel, Linear, and RBF SVMs were compared using nested participant-grouped cross-validation across DE, PLV, and DE–PLV feature representations. For valence, the quantum-kernel SVM using DE features achieved the highest mean balanced accuracy of 59.18%. For arousal, the Linear SVM using DE features achieved the highest mean balanced accuracy of 54.16%. Overall, the three classifiers showed comparable performance across the evaluated feature representations. These results support the feasibility of state-fidelity-based quantum similarity for subject-independent EEG emotion recognition.

## 1. Introduction

Electroencephalography (EEG)-based emotion recognition has been widely studied in affective computing because EEG provides a direct measure of neural activity and has potential applications in adaptive human–computer interaction and affective brain–computer interfaces. The DEAP database is one of the most widely used benchmark datasets for this task [1]. Despite substantial progress, cross-subject emotion recognition remains challenging because EEG signals are non-stationary and exhibit considerable inter-subject variability [2]. Recent evidence further suggests that participant identity can strongly confound EEG emotion-recognition performance when training and test sets contain data from the same individuals, emphasizing the importance of participant-independent evaluation [3]. Under subject-independent evaluation, representative conventional feature-based studies have reported accuracies in the approximately 60–70% range [4,5], while more recent deep-learning and domain-adaptation approaches have reported higher performance [2,6]. For example, Geda et al. [7] proposed EmoDLNet, an end-to-end architecture for learning multi-scale spatio-temporal representations from EEG signals. Beyond emotion recognition, multimodal EEG studies have also explored cross-subject modeling; Geda et al. [8] integrated EEG and eye-tracking signals using cross-modal attention and domain adaptation for preference prediction in immersive VR environments. These developments highlight the importance of both feature representation and evaluation protocol.

Another source of variation among DEAP studies is label construction. While many studies dichotomize valence and arousal ratings around the midpoint, others exclude ambiguous samples near the decision boundary. For example, the STILN subject-independent study classified scores 1–4 as low and 6–9 as high under leave-one-subject-out evaluation [9]. Following the same margin principle, the present study treats ratings ≤4 as low and ratings ≥6 as high, while excluding intermediate ratings.

Emotion-related EEG information can be represented through both local spectral activity and interactions among brain regions. Differential entropy (DE) characterizes band-specific activity at individual EEG channels [10], whereas phase-locking value (PLV) characterizes phase synchronization between channel pairs and provides functional-connectivity information. DE was selected as the spectral descriptor because it provides a compact representation of band-specific EEG activity and has been widely used in EEG emotion recognition, while PLV was included to capture complementary connectivity information that is not directly represented by spectral features such as power spectral density (PSD). Previous work has shown that power-distribution features, including DE, can be combined with PLV-derived functional-connectivity features to represent complementary aspects of emotional EEG activity [11]. However, such fusion also produces a high-dimensional feature space. Therefore, F-score-based feature selection was used in this study to obtain a compact DE–PLV representation for classification.

Beyond feature selection, classification performance also depends on how similarity between samples is represented. Quantum-kernel learning encodes classical features into quantum states and defines pairwise similarity through state fidelity [12,13]. Quantum-kernel methods have also been investigated in application domains beyond EEG, including high-energy physics and materials science [14,15]. The resulting similarity structure is determined by the quantum feature map and its relationship with the input representation.

From a geometric perspective, Huang et al. [16] showed that the relationship between quantum and classical kernel representations can influence predictive performance and the potential for a quantum advantage. Bowles et al. [17] further demonstrated the importance of careful experimental design and meaningful classical benchmarks when evaluating quantum machine-learning models.

Recent studies have begun to explore quantum machine learning for EEG analysis, including its application to emotion recognition, although the role of quantum processing differs substantially among existing approaches. Ho and Hung [18] explored quantum machine learning for EEG classification, providing an early investigation of QML in EEG analysis. Garg et al. [19] formulated SVM training as a quadratic unconstrained binary optimization problem and employed a D-Wave quantum annealer using handcrafted spectral EEG features. Chandanwala et al. [20] proposed a hybrid quantum–classical approach in which EEG frequency-band power features were mapped to quantum representations using rotation and entangling gates before classification with a classical deep-learning model. More recently, Bhatt et al. [21] investigated quantum-kernel-based machine learning for EEG emotion recognition. Yoshida et al. [22] proposed a parallel hybrid architecture combining CNN-derived deep features with quantum-derived features generated from frequency-band representations using a ZZFeatureMap. Their model was evaluated under a segment-level subject-dependent protocol.

These studies demonstrate growing interest in quantum representations for EEG emotion analysis, but differ substantially in feature representation, quantum implementation, and evaluation protocol. In particular, relatively limited attention has been given to state-fidelity quantum kernels constructed from complementary spectral and functional-connectivity EEG features under participant-independent evaluation.

Accordingly, this study investigates the feasibility of state-fidelity-based quantum-kernel learning for subject-independent EEG emotion recognition using a compact DE–PLV representation. Selected EEG features are encoded into quantum states, and pairwise state fidelity is used as the kernel for SVM classification. The quantum-kernel SVM is compared with Linear and RBF SVMs using the same participant partitions, input features, and preprocessing under a nested participant-grouped evaluation framework. Model-specific hyperparameters are selected using only the outer-training participants. The objective is to examine whether the similarity induced by the quantum feature map provides a viable kernel representation for cross-subject EEG emotion recognition, rather than to demonstrate computational quantum advantage.

## 2. Materials and Methods

### 2.1. Dataset and Classification Tasks

The experiments used the preprocessed Python version of the DEAP dataset [1], rather than the raw BDF recordings. The DEAP preprocessing procedure includes downsampling to 128 Hz, band-pass filtering from 4 to 45 Hz, ocular-artifact correction, and re-referencing to the common average. Each trial contains a 3 s pre-stimulus baseline followed by a 60-s stimulus recording.

The first 32 EEG channels from all 32 participants were used, with 40 trials per participant. Valence and arousal were considered as separate binary classification tasks. Ratings ≤4 were assigned to the low class and ratings ≥6 to the high class, while intermediate ratings were excluded. This resulted in 913 valence trials (367 low and 546 high) and 908 arousal trials (376 low and 532 high) as Table 1.

### 2.2. Overview of the Experimental Pipeline

**Figure 1:**
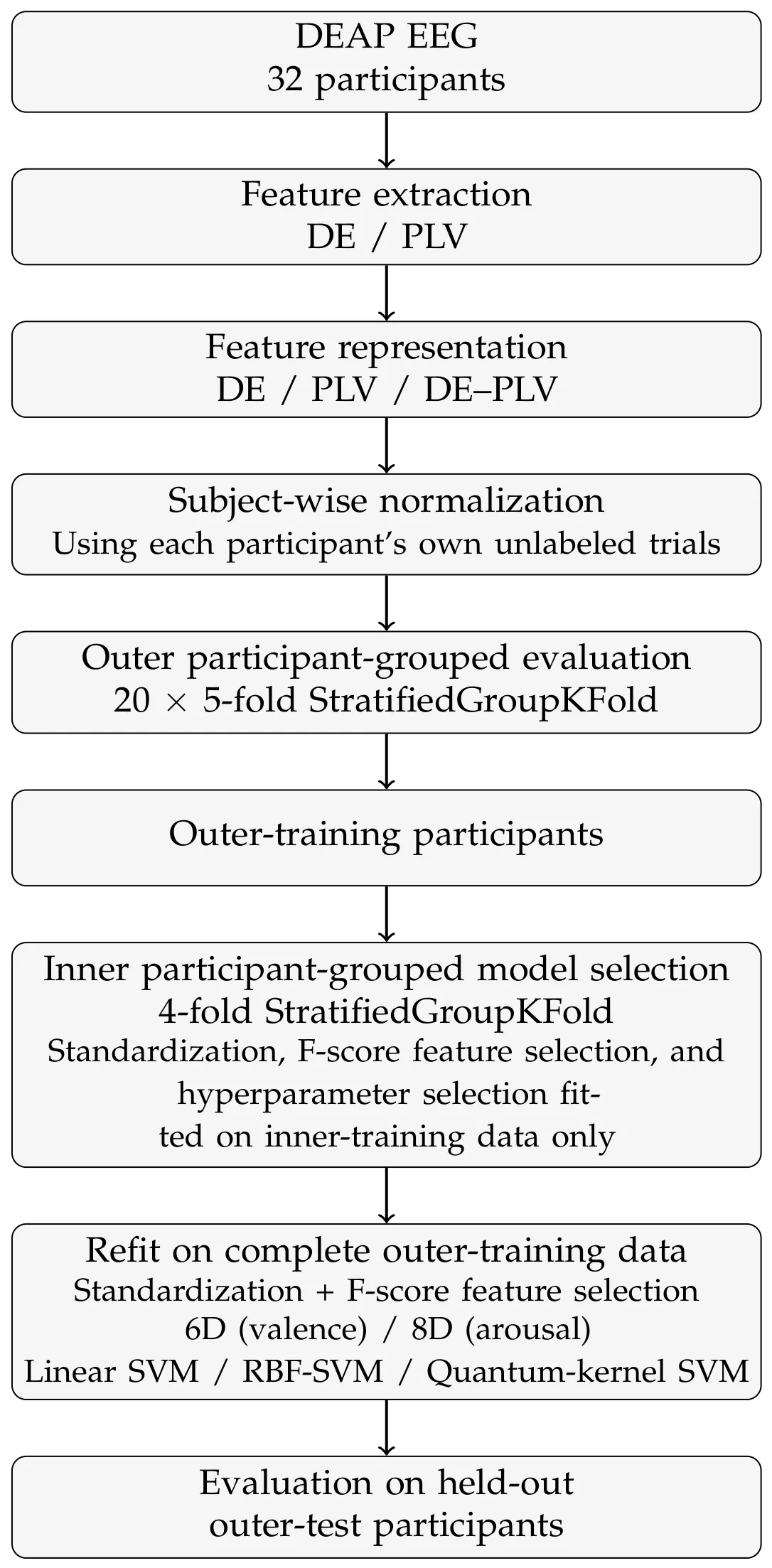
Overview of the nested participant-grouped evaluation framework. Subject-wise normalization was performed separately for each participant using that participant’s own unlabeled trials. The outer evaluation comprised 20 repetitions of five-fold participant-grouped cross-validation. Within each outer-training set, standardization, F-score feature selection, and hyperparameter selection were performed using training data only, with hyperparameters selected through four-fold participant-grouped inner cross-validation. After model selection, standardization and feature selection were refitted on the complete outer-training data, and the resulting models were evaluated on the held-out outer-test participants.

Figure 1 summarizes the overall experimental pipeline. DE and PLV features were extracted from the preprocessed EEG signals to construct three feature representations: DE, PLV, and their concatenation (DE–PLV). Subject-wise normalization was performed independently for each participant using participant-specific feature statistics. Within each outer cross-validation fold, standardization and F-score feature selection were fitted using only the outer-training data, retaining six features for valence and eight for arousal.

For each feature representation, the same selected feature subset was supplied to Linear SVM, RBF-SVM, and quantum-kernel SVM. For the quantum-kernel SVM, the selected features were encoded using a ZFeatureMap with one feature assigned to each qubit, and the resulting state-fidelity kernel was used for SVM classification. Model-specific hyperparameters were selected by inner participant-grouped cross-validation using only the outer-training participants. The selected models were subsequently refitted on the complete outer-training set and evaluated on the held-out participants.

The outer evaluation used 20 repetitions of five-fold StratifiedGroupKFold, with participant identity used as the grouping variable to ensure that the training and test sets contained mutually exclusive participants.

### 2.3. DE–PLV Feature Extraction and Preprocessing

Differential entropy (DE) and phase-locking value (PLV) were extracted to characterize channel-wise spectral activity and inter-channel phase synchronization, respectively. For each trial, the initial 3 s baseline was removed and the complete 60 s stimulus segment from the first 32 EEG channels was used for feature extraction without further temporal segmentation. No additional artifact rejection, ICA, or re-referencing was performed beyond the preprocessing already applied in the DEAP preprocessed dataset.

The 60 s EEG signals were further filtered into four frequency bands using a fourth-order zero-phase Butterworth band-pass filter: theta (4–8 Hz), alpha (8–13 Hz), beta (13–30 Hz), and gamma (30–45 Hz). Filtering was implemented using sosfiltfilt with its default boundary handling, and no additional samples were removed at the filtering boundaries.

For a Gaussian variable with variance $\sigma^{2}$, DE is defined as

(1) $$h\left(X\right)=\frac{1}{2}\ln\left(2\pi e\sigma^{2}\right).$$

DE was calculated for 32 channels in four frequency bands, yielding 128 features per trial. For PLV, instantaneous phase was obtained using the Hilbert transform, and synchronization between channels *i* and *j* was calculated as

(2) $${\mathrm{PLV}}_{ij}=\left|\frac{1}{T}\sum_{t=1}^{T}\exp\left(i[\phi_{i}\left(t\right)-\phi_{j}\left(t\right)]\right)\right|.$$

PLV was calculated for 496 unique channel pairs in four frequency bands, yielding 1984 features per trial. Previous studies have combined local spectral or activation features with PLV-based connectivity for EEG emotion recognition [23,24,25]. Motivated by these studies, DE and PLV were concatenated to form a 2112-dimensional representation containing both band-specific spectral activity and inter-channel phase-synchronization information.

Subject-wise normalization was applied independently to each feature:

(3) $${\tilde{x}}_{s,j}=\frac{x_{s,j}-\mu_{s,j}}{\sigma_{s,j}},$$

where $\mu_{s,j}$ and $\sigma_{s,j}$ denote the mean and standard deviation of feature *j* for participant *s*. These statistics were calculated from all 40 unlabeled trials of each participant before label-based trial exclusion. Thus, held-out participants were normalized using their own unlabeled trials without using emotion labels. This setting assumes that multiple unlabeled trials from a new participant are available before classification.

After subject-wise normalization, F-score-based feature selection was performed using only the training participants within each evaluation split. For a feature *j*, the F-score was calculated as the ratio of between-class variance to within-class variance,

(4) $$F_{j}=\frac{{\left(\mu_{j}^{\left(+\right)}-\mu_{j}\right)}^{2}+{\left(\mu_{j}^{\left(-\right)}-\mu_{j}\right)}^{2}}{\frac{1}{n_{+}-1}\sum_{i=1}^{n_{+}}{\left(x_{i,j}^{\left(+\right)}-\mu_{j}^{\left(+\right)}\right)}^{2}+\frac{1}{n_{-}-1}\sum_{i=1}^{n_{-}}{\left(x_{i,j}^{\left(-\right)}-\mu_{j}^{\left(-\right)}\right)}^{2}},$$

where $\mu_{j}$, $\mu_{j}^{\left(+\right)}$, and $\mu_{j}^{\left(-\right)}$ denote the overall, positive-class, and negative-class means of feature *j*, respectively, and $n_{+}$ and $n_{-}$ are the corresponding numbers of samples. Features with larger F-scores were ranked higher. The resulting feature ranking was then applied to the held-out participants.

### 2.4. Quantum-Kernel and Classical SVM Models

**Figure 2:**
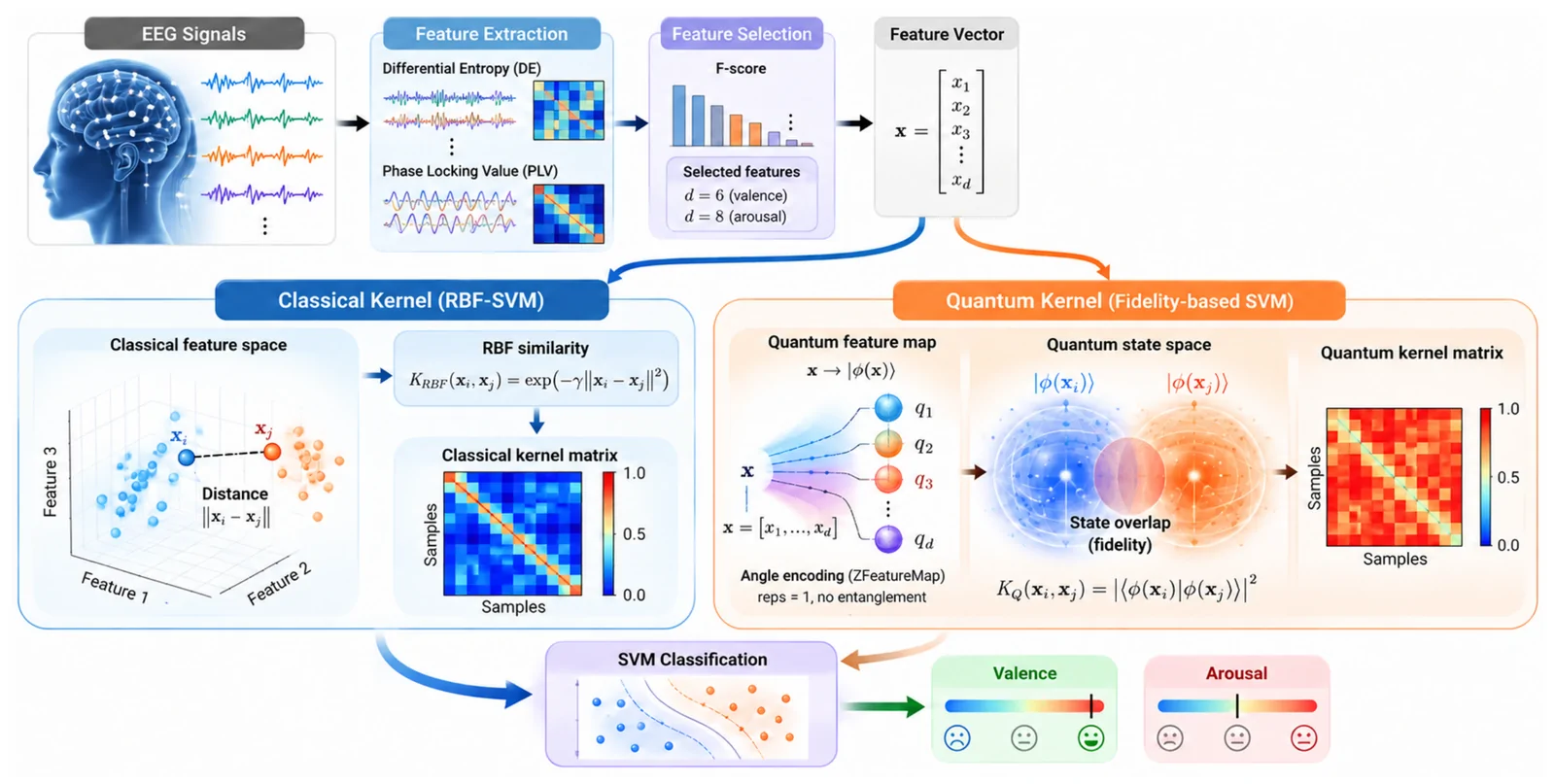
Conceptual comparison of classical and quantum kernel construction for EEG emotion recognition. Classical kernels evaluate relationships between samples directly from the selected EEG feature vectors, whereas the quantum kernel maps the feature vectors to quantum states and evaluates their similarity using state fidelity. The resulting kernel representations are used for SVM-based valence and arousal classification. The arrows indicate the data processing flow. Blue and orange represent the classical and quantum kernel branches, respectively. The symbols $x_{i}$ and $x_{j}$ denote different feature vectors, while $q_{1}$ to $q_{d}$ represent qubits. The ellipses indicate omitted intermediate elements for simplicity and do not affect the scientific interpretation of the figure.

Figure 2 illustrates the conceptual difference between the classical and quantum kernel constructions considered in this study. Classical kernels quantify relationships between samples directly from the selected feature vectors, whereas the quantum kernel first maps the feature vectors to quantum states and evaluates their similarity through state fidelity. In both cases, the resulting kernel representation is used for SVM-based classification.

The quantum-kernel method was implemented using Qiskit (version 2.4.2) and Qiskit Machine Learning (version 0.9.0) [26]. Selected EEG features were encoded using a ZFeatureMap with a single repetition (reps=1) and no entangling operations. Each selected feature was assigned to one qubit; therefore, the six features retained for valence and the eight features retained for arousal were encoded using six- and eight-qubit feature maps, respectively. This configuration was adopted as a baseline for evaluating state-fidelity-based quantum similarity rather than for optimizing quantum circuit architecture.

Before quantum encoding, each selected feature was rescaled to [0,1] using the 2nd and 98th percentiles estimated exclusively from the corresponding training fold. For feature *j*, the lower and upper scaling bounds were defined as

(5) $$l_{j}=P_{2,j}\left(X_{\mathrm{train}}\right),\;h_{j}=P_{98,j}\left(X_{\mathrm{train}}\right),$$

and the feature value was transformed as

(6) $${\tilde{x}}_{j}=\mathrm{clip}\left(\frac{x_{j}-l_{j}}{h_{j}-l_{j}},0,1\right).$$

The same training-derived scaling parameters were applied to the corresponding validation or test data. The rescaled feature was then mapped to a rotation angle according to

(7) $$\theta_{j}=\pi s{\tilde{x}}_{j},$$

where the angle-scaling factor *s* was selected from {0.10,0.25} through inner cross-validation. Thus, percentile scaling and selection of the angle-scaling factor were performed as two distinct stages of the quantum feature-encoding procedure.

For a selected feature vector $x=(x_{1},\ldots ,x_{d})$, where d=6 for valence and d=8 for arousal, the resulting angle vector $\theta =(\theta_{1},\ldots ,\theta_{d})$ was supplied to the ZFeatureMap, with one angle assigned to each corresponding qubit. The feature-to-state mapping was expressed as

(8) $$|\phi \left(x\right)\rangle =U_{\mathrm{ZFM}}\left(\theta \right){|0\rangle }^{⊗d},$$

where $U_{\mathrm{ZFM}}\left(\theta \right)$ denotes the unitary transformation implemented by the ZFeatureMap. Each *d*-dimensional EEG feature vector was therefore represented by a *d*-qubit quantum state.

For two samples $x_{i}$ and $x_{j}$, their quantum similarity was defined by the fidelity between the corresponding encoded states:

(9) $$K_{Q}\left(x_{i},x_{j}\right)={\left|\langle \phi \left(x_{i}\right)|\phi \left(x_{j}\right)\rangle \right|}^{2}.$$

Pairwise fidelity values form the quantum kernel matrix used by the SVM classifier. The kernel was evaluated using FidelityStatevectorKernel on a classical computer; no quantum hardware was used. Accordingly, the present implementation evaluates state-fidelity-based quantum similarity for EEG classification and does not address computational quantum advantage.

Linear and RBF SVMs were included as classical reference models. The linear kernel evaluates similarity through the inner product between feature vectors, whereas the RBF kernel defines similarity as

(10) $$K_{\mathrm{RBF}}\left(x_{i},x_{j}\right)=\exp\left(-\gamma {∥x_{i}-x_{j}∥}^{2}\right).$$

To account for class imbalance, all three SVM classifiers used class weights inversely proportional to the class frequencies in the corresponding training data (class_weight="balanced"). No resampling or decision-threshold adjustment was applied.

All three classifiers used identical participant splits, subject-wise normalization, and F-score-selected input features. Model-specific hyperparameters were selected within the same inner participant-grouped cross-validation framework, as described in Section 2.5.

### 2.5. Nested Participant-Grouped Evaluation

Performance was evaluated using nested participant-grouped cross-validation. The outer evaluation comprised 20 repetitions of five-fold StratifiedGroupKFold, with participant identity as the grouping variable, yielding 100 subject-separated test folds per emotion dimension and classifier. To ensure reproducibility, random seeds from 1000 to 1019 were used for the 20 repeated outer cross-validation runs. For each outer fold, the seed for inner cross-validation was deterministically derived from the corresponding outer-run seed and fold index.

The evaluation was performed separately for three feature representations: DE, PLV, and the concatenated DE–PLV representation. For each representation, feature selection and model training were performed independently using the same participant splits and evaluation procedure.

Within each outer-training set, hyperparameters were selected using four-fold StratifiedGroupKFold restricted to the outer-training participants. Within each inner fold, feature standardization and F-score calculation were fitted exclusively on the inner-training data and then applied to the corresponding inner-validation data. After hyperparameter selection, standardization and feature selection were refitted using the complete outer-training set and applied to the held-out outer-test data. This training-based standardization was distinct from the preceding subject-wise normalization, which used each participant’s own unlabeled trials as described in Section 2.5. Outer-test participants were not used for feature selection, hyperparameter selection, or model training.

The hyperparameter search grids were defined as follows. For Linear SVM, the regularization parameter was searched over C∈{0.1,1,10}. For RBF-SVM, the search grid was C∈{0.1,1,10} and γ∈{scale,0.01,0.1,1.0}. For the quantum-kernel SVM, the search grid was C∈{0.1,1,10} and the angle-scaling factor was s∈{0.10,0.25}. Balanced accuracy (BA) was used as the hyperparameter-selection criterion within the inner participant-grouped cross-validation.

Because each selected feature corresponds to one qubit in the quantum feature map, a compact feature representation was used. Preliminary feature-dimension comparisons considered 4, 6, 8, and 10 top-ranked features to balance representation capacity with the computational cost associated with increasing the number of qubits. Among the evaluated dimensions, six features yielded the best performance for valence and eight for arousal. These dimensions were therefore fixed for the subsequent comparative experiments.

Feature selection was performed independently for each of the DE, PLV, and DE–PLV representations, and the same selected features were supplied to all three classifiers within each outer split for a given representation. For the quantum-kernel SVM, each selected feature corresponded to one qubit, resulting in six- and eight-qubit circuits for valence and arousal, respectively.

BA was used as the primary metric because of class imbalance:

(11) $$\mathrm{BA}=\frac{1}{2}\left(\frac{\mathrm{TP}}{\mathrm{TP}+\mathrm{FN}}+\frac{\mathrm{TN}}{\mathrm{TN}+\mathrm{FP}}\right).$$

Accuracy (ACC) and macro-averaged F1-score (Macro-F1) were additionally reported. Performance distributions were characterized using the 100 outer-fold scores.

## 3. Results

### 3.1. Classification Performance

Table 2 summarizes the classification performance across the three feature representations and three classifiers. For valence, DE-based models generally achieved higher performance than the corresponding PLV and DE–PLV models. The highest mean BA was obtained by the quantum-kernel SVM using DE features (59.18%), while the Linear SVM using DE achieved the highest ACC (58.59%) and Macro-F1 (58.09%).

For arousal, the differences across feature representations and classifiers were smaller. The Linear SVM using DE achieved the highest mean BA (54.16%), whereas the quantum-kernel SVM using PLV achieved the highest ACC (54.14%) and Macro-F1 (53.32%). Overall, no single classifier consistently achieved the best performance across feature representations, emotion dimensions, and evaluation metrics.

**Figure 3:**
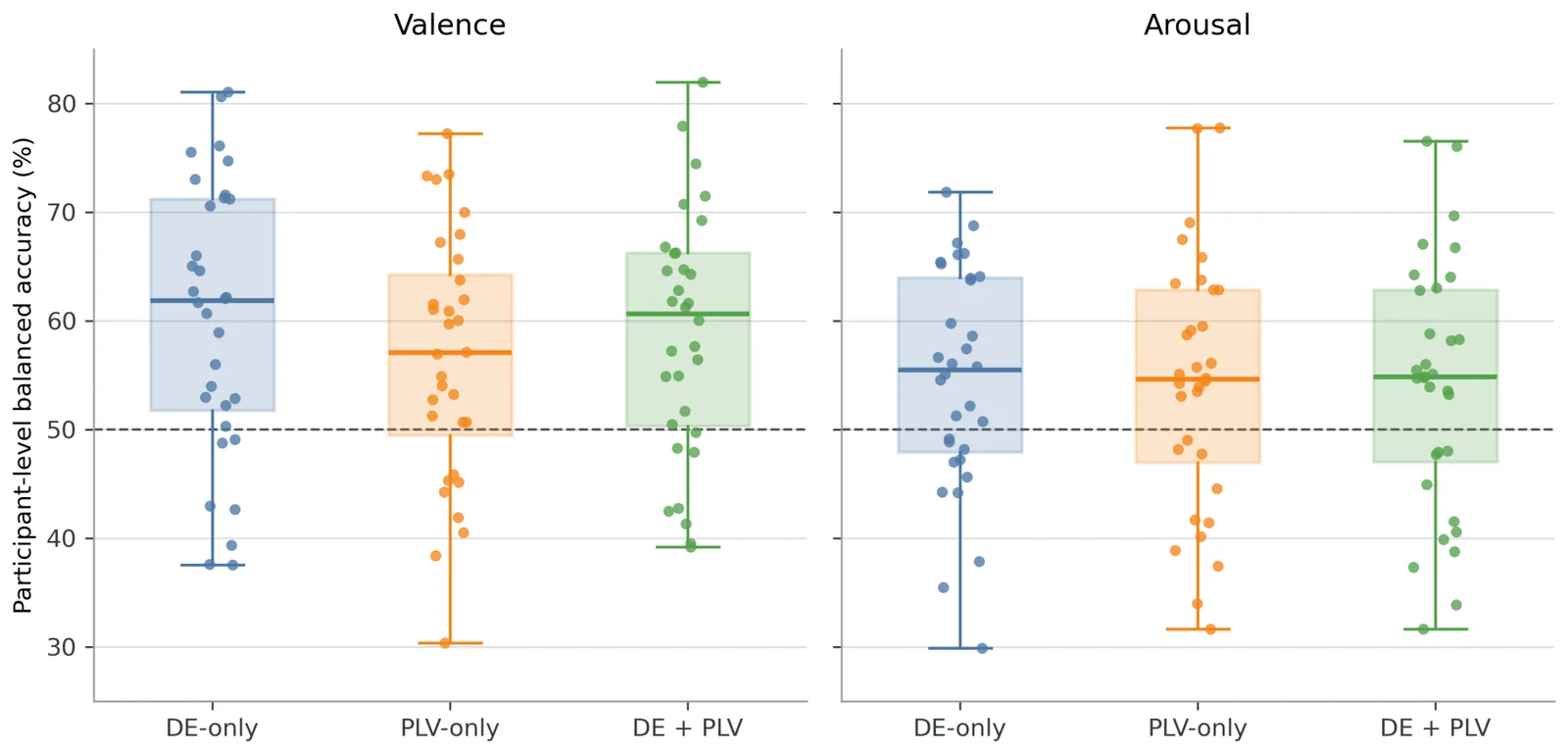
Participant-level balanced accuracy of the quantum-kernel SVM across DE-only, PLV-only, and combined DE–PLV feature representations for valence and arousal. Each point represents the mean balanced accuracy of one participant across the 20 repeated outer cross-validation runs. Boxes indicate the interquartile range, center lines indicate the median, and whiskers indicate the observed range. The dashed horizontal line denotes the 50% chance level.

To further examine inter-subject variability, participant-level performance of the quantum-kernel SVM was analyzed across the three feature representations. As shown in Figure 3, noticeable inter-subject variability was observed for both emotion dimensions. For valence, the DE representation showed a generally higher participant-level performance distribution than PLV and DE–PLV, consistent with the aggregate results in Table 2. For arousal, the participant-level distributions were more similar across the three feature representations.

**Figure 4:**
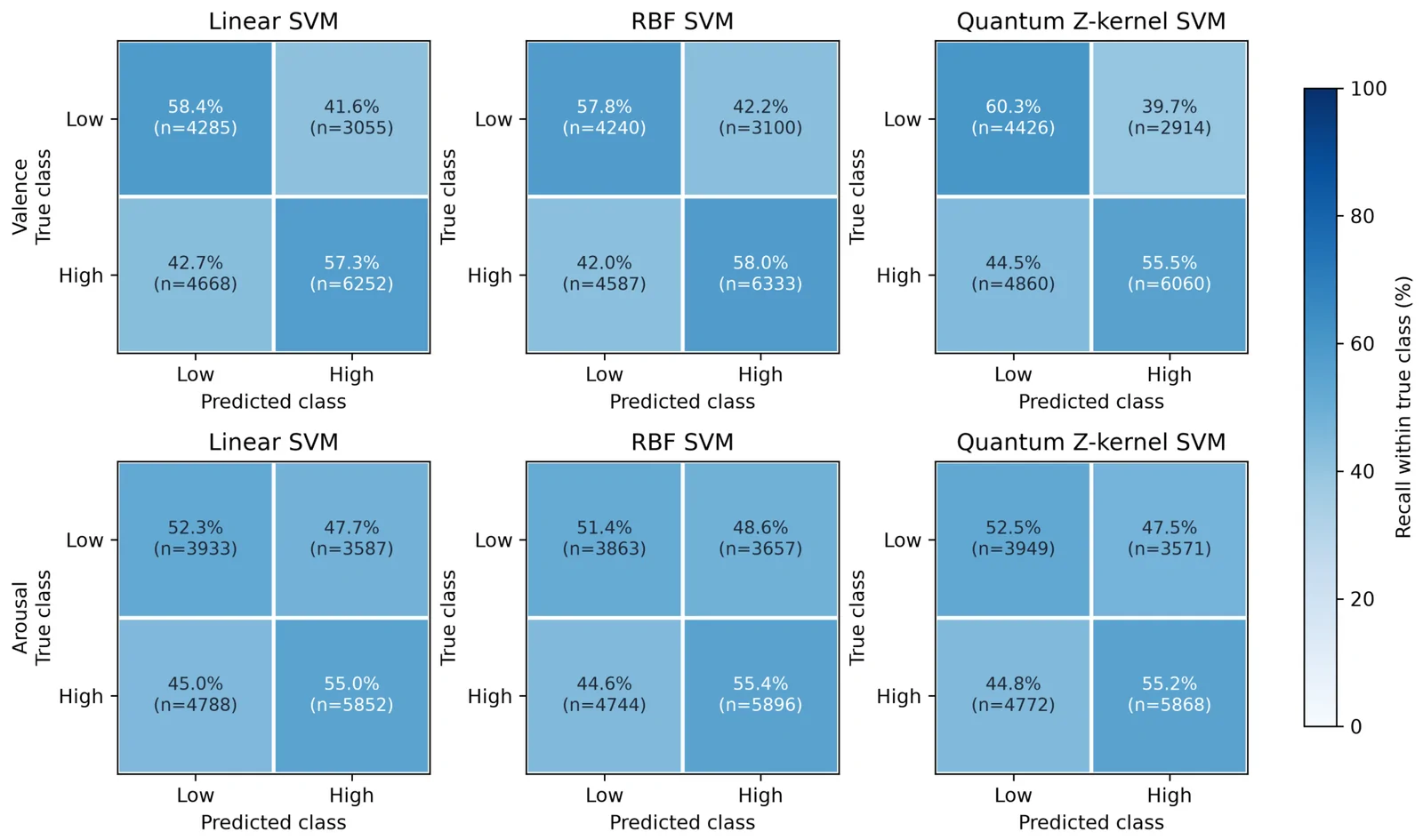
Row-normalized confusion matrices for the Linear SVM, RBF-SVM, and quantum-kernel SVM using the combined DE–PLV feature representation for valence (top row) and arousal (bottom row). Predictions were aggregated across the 20 repeated outer cross-validation runs. Each cell shows the percentage of predictions within the corresponding true class, with the aggregated number of predictions shown in parentheses.

Class-wise recognition was further examined under the combined DE–PLV representation. As shown in Figure 4, the Linear and RBF SVMs showed relatively balanced recall between the low and high classes for valence, whereas the quantum-kernel SVM showed higher recall for the low class than for the high class. For arousal, all three classifiers showed lower recall for the low class than for the high class.

### 3.2. Feature-Selection Consistency

To examine the consistency of fold-wise feature selection, the selection frequency of each feature was calculated across the 100 outer folds for the DE–PLV representation. Within each outer fold, six features were retained for valence and eight for arousal, with the same selected feature subset supplied to all three classifiers. Table 3 reports the most frequently selected features, corresponding to the number of features retained for each emotion dimension. A selection frequency of, for example, 56/100 indicates that the corresponding feature was retained in 56 of the 100 outer folds.

Several features were repeatedly selected despite feature selection being performed independently within each outer-training fold. For valence, DE–α–AF3 and PLV–α–FC6–Cz were each selected in 84 of the 100 outer folds, followed by DE–β–Pz in 67 folds and DE–α–P4 in 58 folds. For arousal, PLV–β–F3–Cz showed particularly high selection consistency, being selected in 99 of the 100 outer folds. The remaining high-frequency features were predominantly β-band PLV features. Although the complete selected feature subsets varied across participant partitions, several features were repeatedly retained.

Across the ablation results, feature representation had a more noticeable effect on mean classification performance than classifier choice, particularly for valence.

### 3.3. Computational Cost

The computational cost was evaluated using the same nested cross-validation setting as in the main experiments. Runtime was measured on an Apple M2 Max system with a 12-core CPU and 64 GB of memory. The measurements included inner-loop hyperparameter tuning and final outer-fold model fitting and prediction. The quantum kernel was evaluated using exact statevector simulation in Qiskit.

For valence, the mean runtime per outer fold was 0.056 s for the Linear SVM, 0.429 s for the RBF-SVM, and 15.76 s for the quantum-kernel SVM. For arousal, the corresponding runtimes were 0.053 s, 0.441 s, and 17.42 s, respectively. Under the present simulation-based setting, the quantum-kernel implementation required approximately 37–40 times the runtime of the RBF-SVM. Profiling further showed that approximately 99% of the quantum-model runtime was associated with quantum kernel-matrix evaluation.

## 4. Discussion

The present study investigated the feasibility of state-fidelity-based quantum-kernel learning for subject-independent EEG emotion recognition and examined its performance across DE, PLV, and combined DE–PLV feature representations.

Under the nested participant-grouped evaluation, the quantum-kernel SVM showed performance broadly comparable to the classical SVMs across the three feature representations, while the best-performing classifier varied across feature representations, emotion dimensions, and evaluation metrics. These results indicate that the quantum-kernel SVM can provide classification performance comparable to established classical kernels, but do not demonstrate a consistent performance advantage.

The feature-representation ablation further showed that classification performance depended on the input representation. For valence, DE alone yielded higher mean BA than PLV or the concatenated DE–PLV representation across all three classifiers. For arousal, the differences among feature representations were smaller, and the best-performing combinations varied across evaluation metrics. In particular, concatenating DE and PLV did not consistently improve classification performance. Although DE and PLV characterize complementary aspects of EEG activity, representing local spectral activity and inter-channel phase synchronization, respectively, their combination did not necessarily translate into improved predictive performance after feature selection. Overall, the results suggest that the choice of feature representation had a more noticeable influence on performance than classifier choice, particularly for valence.

The participant-level analysis further showed noticeable inter-subject variability across the evaluated feature representations, indicating that aggregate performance metrics do not fully reflect differences in classification difficulty across individuals. This variability is consistent with the challenge of subject-independent EEG emotion recognition, where individual differences in EEG responses can affect classification performance. Class-wise analysis further showed that recognition difficulty was not uniform between the Low and High classes. In particular, for arousal, lower recall for the Low class was observed across all three classifiers under the DE–PLV representation. These results highlight participant- and class-dependent variability that should be considered when interpreting the aggregate classification performance.

The comparisons were conducted using the same participant partitions, normalization procedure, feature-selection procedure, and nested model-selection framework. For each feature representation, F-score selection was performed independently within the training data, and the same selected features were supplied to all three classifiers. Classifier-specific hyperparameters were selected only within the outer-training participants, reducing the possibility that performance differences resulted from unequal opportunities for optimization. Six features were retained for valence and eight for arousal, corresponding to six- and eight-qubit feature maps for the quantum model. Thus, the results support the feasibility of state-fidelity-based quantum similarity using compact EEG feature representations, but do not indicate that the quantum kernel requires fewer features or provides superior predictive performance relative to the classical reference models.

The present study employed a simple ZFeatureMap with a single repetition and no entangling operations. The resulting product-state encoding can be efficiently evaluated classically, and no computational quantum advantage is claimed for the present implementation. The configuration should therefore be interpreted as a simple quantum-kernel baseline for examining state-fidelity-based similarity in EEG emotion recognition rather than as a classically intractable representation. Different circuit structures, repetition depths, encoding scales, and entanglement patterns may induce different kernel geometries.

The interpretation of the present results should also consider the evaluation protocol. Although training and test participants were strictly separated within each outer fold, the repeated outer folds are not statistically independent because participants can appear in different test folds across repetitions. The repeated evaluation was therefore used to characterize performance stability across participant partitions rather than to increase the effective sample size. In addition, the exclusion of intermediate affective ratings may affect task difficulty and generalisability to settings that include intermediate or ambiguous affective states. Because alternative labeling strategies were not directly compared in the present study, the magnitude of these effects cannot be quantified from the current results. Absolute performance should therefore be compared with caution across studies using different labeling strategies.

Future work will extend the present study in several directions. The proposed framework will be evaluated on additional EEG emotion datasets and under alternative labeling strategies, including an examination of the influence of intermediate ratings on classification performance. Complementary subject-independent evaluation protocols, such as leave-one-subject-out validation, will also be considered. In addition, more expressive quantum feature maps with deeper circuits and entangling operations will be investigated, together with their behavior under realistic quantum-hardware conditions.

## 5. Conclusions

This study investigated the feasibility of state-fidelity-based quantum-kernel learning for subject-independent EEG emotion recognition across DE, PLV, and combined DE–PLV feature representations. F-score feature selection provided compact six- and eight-dimensional inputs for valence and arousal, respectively, which were supplied to Linear SVM, RBF-SVM, and quantum-kernel SVM under the same nested participant-grouped evaluation framework.

The results showed comparable classification performance across the three classifiers, while the best-performing combination varied across feature representations, emotion dimensions, and evaluation metrics. Feature representation had a more noticeable influence on mean performance than classifier choice, particularly for valence, and combining DE and PLV did not consistently improve performance. Overall, the results support the feasibility of state-fidelity-based quantum similarity as a classification kernel for subject-independent EEG emotion recognition.

Future work should investigate alternative quantum feature maps, including different circuit structures, repetition depths, and entanglement patterns, and should validate the approach using independent EEG datasets and quantum hardware.

## Figures and Tables

**Table 1:** Dataset and binary emotion-classification tasks used in this study.

Task	Total Trials	Low Emotion	High Emotion
Valence	913	367	546
Arousal	908	376	532

**Table 2:** Classification performance across feature representations and classifiers under the nested participant-grouped evaluation protocol. Values are reported as mean ± standard deviation. BA denotes balanced accuracy, ACC denotes accuracy, and Macro-F1 denotes macro-averaged F1-score.

Feature	Classifier	BA (%)	ACC (%)	Macro-F1 (%)
**(a) Valence**				
DE	Linear SVM	58.91 ± 4.96	**58.59 ± 4.83**	**58.09 ± 4.80**
	RBF-SVM	58.83 ± 5.22	58.05 ± 5.02	57.64 ± 5.03
	Quantum-kernel SVM	**59.18 ± 5.23**	58.36 ± 4.98	57.96 ± 5.02
PLV	Linear SVM	55.90 ± 4.88	55.81 ± 4.75	55.24 ± 4.73
	RBF-SVM	54.87 ± 4.94	55.38 ± 5.20	54.20 ± 5.36
	Quantum-kernel SVM	55.71 ± 4.96	55.70 ± 5.01	55.03 ± 4.89
DE + PLV	Linear SVM	57.79 ± 5.01	57.67 ± 4.82	57.10 ± 4.85
	RBF-SVM	57.92 ± 4.85	57.89 ± 4.82	57.04 ± 4.87
	Quantum-kernel SVM	57.81 ± 5.39	57.37 ± 5.21	56.87 ± 5.21
**(b) Arousal**				
DE	Linear SVM	**54.16 ± 3.15**	53.42 ± 3.26	53.19 ± 3.27
	RBF-SVM	53.21 ± 3.17	51.71 ± 3.51	51.34 ± 3.57
	Quantum-kernel SVM	53.42 ± 3.40	51.45 ± 3.90	51.23 ± 3.94
PLV	Linear SVM	53.60 ± 4.48	53.85 ± 4.54	53.22 ± 4.51
	RBF-SVM	53.53 ± 4.41	54.04 ± 4.68	52.93 ± 5.06
	Quantum-kernel SVM	53.81 ± 4.50	**54.14 ± 4.67**	**53.32 ± 4.61**
DE + PLV	Linear SVM	53.57 ± 4.32	53.81 ± 4.38	53.18 ± 4.35
	RBF-SVM	53.33 ± 4.50	53.68 ± 4.80	52.48 ± 5.56
	Quantum-kernel SVM	53.68 ± 4.44	53.97 ± 4.60	53.18 ± 4.55

Note: Bold values indicate the best performance in each comparison.

**Table 3:** Most frequently selected features across the 100 outer folds for the DE–PLV representation. Six features were retained per fold for valence and eight for arousal.

Task	Feature	Selection Frequency
Valence	DE–α–AF3	84/100
	PLV–α–FC6–Cz	84/100
	DE–β–Pz	67/100
	DE–α–P4	58/100
	DE–α–FC6	45/100
	PLV–α–F3–Cz	41/100
Arousal	PLV–β–F3–Cz	99/100
	PLV–β–F3–P3	56/100
	PLV–β–P3–CP2	55/100
	PLV–β–Fp1–P3	52/100
	PLV–γ–F3–F4	44/100
	PLV–β–Fp1–Cz	43/100
	PLV–β–T7–P3	37/100
	PLV–β–P3–Fp2	33/100

## Data Availability

The DEAP dataset analyzed in this study is available from the DEAP project website subject to its data-access conditions. No new EEG dataset was generated in this study.

## Abbreviations

The following abbreviations are used in this manuscript:

ACC	Accuracy
BA	Balanced Accuracy
DE	Differential Entropy
DEAP	Database for Emotion Analysis Using Physiological Signals
EEG	Electroencephalography
Macro-F1	Macro-Averaged F1-Score
PLV	Phase-Locking Value
QML	Quantum Machine Learning
RBF	Radial Basis Function
SVM	Support Vector Machine
